# RNA profiles differ between small and large extracellular vesicle subsets isolated from porcine seminal plasma

**DOI:** 10.1186/s12864-024-11167-4

**Published:** 2024-12-27

**Authors:** Isabel Barranco, Carmen Almiñana, Ana Parra, Pablo Martínez-Diaz, Xiomara Lucas, Stefan Bauersachs, Jordi Roca

**Affiliations:** 1 Department of Medicine and Animal Surgery, Veterinary Science, University of Murcia, Murcia, Spain; 2 Institute of Veterinary Anatomy, Vetsuisse-Faculty, University of Zurich, Lindau, ZH Switzerland; 3 Department of Reproductive Endocrinology, University Hospital Zurich, Zurich, Switzerland

**Keywords:** Extracellular vesicles, Porcine, RNA, Seminal plasma, Subsets

## Abstract

**Background:**

Extracellular vesicles (EVs) are essential for cell-to-cell communication because they transport functionally active molecules, including proteins, RNA, and lipids, from secretory cells to nearby or distant target cells. Seminal plasma contains a large number of EVs (sEVs) that are phenotypically heterogeneous. The aim of the present study was to identify the RNA species contained in two subsets of porcine sEVs of different sizes, namely small sEVs (S-sEVs) and large sEVs (L-sEVs). The two subsets of sEVs were isolated from 54 seminal plasma samples by a method combining serial centrifugations, size exclusion chromatography, and ultrafiltration. The sEVs were characterized using an orthogonal approach. Analysis of RNA content and quantification were performed using RNA-seq analysis.

**Results:**

The two subsets of sEVs had different size distributions (*P* < 0.001). They also showed differences in concentration, morphology, and specific protein markers (*P* < 0.05). A total of 735 RNAs were identified and quantified, which included: (1) mRNAs, rRNAs, snoRNAs, snRNAs, tRNAs, other ncRNAs (termed as “all RNAs”), (2) miRNAs and (3) piRNAs. The distribution pattern of these RNA classes differed between S-sEVs and L-sEVs (*P* < 0.05). More than half of “all RNAs”, miRNAs and piRNAs were found to be differentially abundant between S- and L-sEVs (FDR < 0.1%). Among the differentially abundant RNAs, “all RNAs” were more abundant in L- than in S-sEVs, whereas the most of the miRNAs were more abundant in S- than in L-sEVs. Differentially abundant piRNAs were equally distributed between S- and L-sEVs. Some of the all RNAs and miRNAs found to be differentially abundant between S- and L-sEVs were associated with sperm quality and functionality and male fertility success.

**Conclusions:**

Small and large sEVs isolated from porcine seminal plasma show quantitative differences in RNA content. These differences would suggest that each sEV subtype exerts different functional activities in the targeted cells, namely spermatozoa and functional cells of the female reproductive tract.

**Supplementary Information:**

The online version contains supplementary material available at 10.1186/s12864-024-11167-4.

## Background

Extracellular vesicles (EVs) are one of the most important mechanisms of cell-to-cell communication [1]. The EVs circulate freely in body fluids and carry biologically active molecules from secretory cells to nearby or distant target cells. Transported molecules include, e.g., proteins, lipids, and RNA that, once released into target cells, regulate their functional activity. Thereby, EVs have been shown to be involved in many physiological and pathological processes, including tissue regeneration [2] and cancer [3].

Circulating EVs in the fluids of male and female reproductive tract would be involved in many of the major physiological reproductive processes, such as fertilization, embryo development, and placentation, but also in pathological reproductive processes such as endometriosis [4, 5]. Seminal plasma (SP), the fluid that surrounds sperm during and after ejaculation, is one of the body fluids with the highest number of EVs [6]. Seminal EVs (sEVs) are released from the functional cells of the testis, epididymis, vas deferens and accessory glands, and deliver their molecular cargo to spermatozoa and functional cells of the female reproductive tract [7]. Thus, they would be involved in sperm functionality, male fertility success, and embryo development [8]. Despite these promising findings, sEVs have been comparatively less studied than those circulating in other biofluids [9], so advancing their understanding remains an outstanding scientific challenge. The population of circulating EVs in SP, like those present in other body fluids, is heterogeneous in size, shape, and EV protein markers [10, 11], and unraveling this heterogeneity is another open challenge for the scientific community [12].

There is growing evidence that sEVs contain a large number of RNAs that may be directly or indirectly involved in sperm functionality and, ultimately, male fertility [13]. Most of the current studies on sEV RNAs have focused on specific RNA species, particularly microRNAs (miRNAs). In pigs, some miRNAs identified in sEVs have been associated with sperm maturation and functionality and male reproductive success [7, 14, 15]. However, it is still unknown whether the identified sEV miRNAs are associated with a specific sEV subset [13]. In addition to miRNAs, sEVs contain many other RNA species that have attracted less research interest, but which also deserve to be explored as they may have functional relevance [16]. Therefore, this study allows the quantification of all RNA species loaded in porcine sEVs, especially small non-coding RNA (sncRNAs) species other than miRNAs, which have recently gained scientific interest [17].

Accordingly, the aim of the present study was to identify and quantify the RNA species contained in two size-differentiated porcine sEV subsets, namely large and small sEVs, two subtypes of EVs recognized by the International Society for Extracellular Vesicles (ISEV) [18]. Large and small porcine sEV subsets show structural and morphological differences that would be defined by the releasing cell source [11, 19]. Thus, small sEVs would be mainly released from epididymal and prostate cells, whereas large sEVs would be mainly released from seminal vesicle cells. Different functional roles have been observed for the two sEV subtypes. For example, in an in vitro fertilization setting, large sEVs have a greater effect on sperm metabolism than small sEVs [20]. Elucidating potential differences in RNA cargo between the two sEV subsets should facilitate understanding of the distinct functional roles of small and large sEVs in reproductive biology.

## Methods

### Animals, ejaculates, and seminal plasma samples

The procedures used to collect semen samples from boars were validated by the Bioethics Committee of the University of Murcia (research codes: CBE 367/2020 and CBE: 538/2023) in accordance with the international guidelines for the protection of animals used for scientific purposes (Directive 2010/63/EU). The semen donor boars belonged to the company AIM Ibérica (Topigs Norsvin España, Madrid, Spain) and were housed in an artificial insemination (AI) center located in Calasparra (Murcia, Spain). The Center complies with Spanish (ES300130640127; August 2006) and European Union (ES13RS04P; July 2012) guidelines on animal welfare and on the collection of ejaculates and the production and marketing of semen AI doses. Boars were housed in individual pens in an air-controlled barn with 16 h of daylight per day. Boars had access to water *ad libitum* and were fed a commercial diet adapted in quality and quantity to meet the nutritional requirements of AI boars in production.

The semen samples used in the experiment were from 54 ejaculates collected from Landrace and Large White boars. These boars were included in a commercial AI program and were therefore subject to a regular ejaculate collection schedule of two ejaculates per week. Entire ejaculates were collected using the semi-automated Collectis^®^ method (IMV Technologies, L’Aigle, France). Semen samples (15 mL) from each of the 54 ejaculates were subjected immediately after ejaculate collection to two consecutive 1,500 xg centrifugations at room temperature (RT) for 10 min (Rotofix 32 A; Hettich Zentrifugen, Tuttlingen, Germany) to remove spermatozoa and any other cellular debris. The resulting SP samples were shipped in a thermal box (5 °C) to the Animal Andrology Laboratory of the University of Murcia (Spain), where they arrived in less than 1 h. In the laboratory, the samples were stored at -80 °C (Ultra Low Freezer; Haier, Schneiderberg, Ontorio, Canada) until isolation of the sEVs.

### Isolation of sEVs

The 54 SP samples were thawed on ice and randomly pooled into 12 biological replicates of 4 or 5 SP samples each. The sEVs were isolated using the size exclusion chromatography (SEC)-based method described by Barranco et al. [21], which allows the separate isolation of two sEV subsets, one with larger sEVs (L-sEVs) and one with smaller sEVs (S-sEVs). In addition, the SEC has been shown to be an optimal method for isolating EV samples for RNA analysis [22], minimizing contamination by non-EV proteins and RNAs [23]. In detail, 4 mL of each of the 12 biological replicates were centrifuged at 3,200 xg (4 °C, 15 min; Sorvall™ STR40, Thermo Fisher Scientific, Waltham, MA, USA) and the resulting supernatants were centrifuged at 20,000 xg (4 °C, 30 min; Sorvall™ Legend™ Micro 21R, Thermo Fisher Scientific). The supernatants were used to isolate S-sEVs and the pellets were used to isolate L-sEVs. For this purpose and prior to SEC, the supernatants were diluted (1:2; v: v) in filtered (0.22 μm, Millex^®^ syringe filters) phosphate buffer saline (PBS), then filtered (0.22 μm, Millex^®^ syringe filters), and ultrafiltered using Amicon^®^ Ultra-4mL 10 kDa centrifugal filters (UFC8003, Merck KGaA, Darmstadt, Germany) and repeated centrifugation cycles at 3,200 xg at 4 °C (approximately 90 min in total). The concentrated sample retained on the ultrafiltration membrane was used for SEC processing. The pellets were directly resuspended with 500 µL of filtered PBS for SEC processing. SEC was performed on home-made columns using 20 mL SEC tubes (Econo-Pac^®^ Chromatography Columns 20 mL, Bio-Rad Laboratories, Hercules, California, USA) loaded with 10 mL Sepharose CL2B^®^ (Sigma Aldrich^®^, Merck KGaA). Twenty 500-µL elution fractions were collected from each SEC column, with fractions 7 through 10 saved as they were the most enriched in sEVs. A single two mL sample was prepared for each SEC by pooling these four eluted fractions. Each resulting 2 mL SEC sample was divided into two aliquots that were either stored at 5 °C (Zanussi Tropic System, Electrolux España, Madrid, Spain) or frozen at–80 °C (Ultra Low Freezer; Haier). The sample stored at 5 °C was used for phenotypic characterization of sEVs, which was performed 24–48 h after sEV isolation. Samples frozen at − 80 °C were shipped on dry ice to the University of Zurich for sEV RNA analysis.

### Characterization of sEVs

Characterization of sEV samples was performed using an orthogonal approach according to the recommendations of the “Minimal information for studies of extracellular vesicles 2023” guideline [18]. Characterization included measurements of EV concentration, particle size distribution, EV morphology, specific EV protein markers, non-vesicular extracellular particle markers, and quantification of total protein in the sEV sample.

The concentration of sEVs was quantified by nanoparticle tracking analysis (NTA) using a NanoSight LM10 according to the manufacturer’s instructions (Malvern Instrument Ltd, Malvern, UK). The NanoSight was equipped with a 405 nm laser and a complementary metal oxide semiconductor scientific chamber. Five measurements were performed per sample. The size distribution of sEVs was analyzed by dynamic light scattering (DLS) using a Zetasizer Nano ZS (Malvern Panalytical, Malvern, UK) operating at 633 nm at RT. Backscattered light was recorded at an angle of 173° and data analysis was performed using Dispersion Technology v.5.10 software (Malvern Panalytical). For analyses, sample volumes were 50 µL, light scattering was recorded for 150 s, and three measurements were taken per sample. The morphology of sEVs was visualized by transmission electron microscopy (TEM) using a JEOL JEM 1011 (JEOL Ltd., Tokyo, Japan) at 80 kV. Samples of 10 µL were fixed with 0.1% paraformaldehyde for 30 min and placed on a carbon-coated copper grid, which was incubated for seven min. Total protein concentration in sEV samples was quantified using the Micro BCA kit according to the manufacturer’s recommendations (Thermo Fisher Scientific).

Specific EV protein markers and non-vesicular extracellular particle markers were analyzed by flow cytometry using a high-sensitivity flow cytometer (CytoFLEX S; Beckman Coulter, Life Sciences Division Headquarters, Indianapolis, USA) according to the protocol described by Barranco et al. [11]. Recombinant EVs expressing green fluorescent protein on the membrane (SAE0193, Merck) were used to verify the accuracy of the flow cytometer for EV gating and analysis. Analysis was restricted to events whose size (forward scatter, FSC) and complexity (violet side scatter (SSC)-A) matched those of the EVs. Samples were analyzed at a low flow rate (5–10 µL/min) with a minimum of 10 × 10^3^ events per sample. For analysis, 10 µL of sEV samples were incubated with CellTrace™ CFSE (carboxyfluorescein succinimidyl ester; Thermo Fisher Scientific) to identify and delimit the analysis to membrane-intact sEVs. The protein EV markers selected were CD63 as a category 1 protein (transmembrane or GPI-anchored proteins associated with the plasma membrane and/or endosomes) and 90 kDa heat shock protein (HSP90β) as a category 2 protein (cytosolic proteins recovered in EVs). The presence of albumin, a category 3 protein (major components of coisolated non-vesicular extracellular particles), was identified to assess the purity of the sEVs samples. The antibodies used were anti-CD63-FITC (Fluorescein isothiocyanate; clone REA1055, Miltenyi Biotec, Bergisch Gladbach, Germany); anti-HSP90β-PE (Phycoerythrin; ADI-SPA-844PE-050, Enzo Life Sciences, Farmingdale, NY, USA); and anti-albumin-FITC (CLFAG16140, Cedarlane, Burlington, Canada, USA).

### Analysis of the RNA content of sEVs

#### Concentration of sEV samples and isolation of RNA

A total of 24 sEV samples were processed for RNA sequencing (RNA-seq), specifically 12 S-sEV and 12 L-sEV samples. The sEV samples were first concentrated by resuspension to 1,750 µL with PBS-trehalose (25 mM) and ultracentrifugation at 100,000 xg for 90 min at 4 °C. The pellets were then resuspended in 20 µL PBS-trehalose [24]. Total RNA was isolated using the miRNeasy micro kit (QIAGEN AG, Hombrechtikon, Switzerland) according to the manufacturer’s instructions. RNA concentration and quality profiles of sEV samples were measured on an Agilent 2100 Bioanalyzer (Agilent Technologies Schweiz AG, Basel, Switzerland) using the Agilent RNA 6000 Pico kit (Agilent Technologies Schweiz AG).

#### Low-input total RNA-seq library preparation and sequencing

A total of 24 libraries were prepared, specifically 12 S-sEV and 12 L-sEV samples, 3 replicates (R)/experimental group. RNA-seq library preparation was performed using the SEQuoia Complete Stranded RNA Library Prep Kit (Bio-Rad Laboratories, Inc., Cressier, Switzerland), which allows the capture of both long and short RNAs in a single library. To remove ribosomal RNA fragments, the QIAseq FastSelect Stranded RNA Removal Kit (QIAGEN AG) was used in the first step of library preparation. A total of 2 ng total RNA was used for L-sEV samples and 0.8 ng for S-sEV samples. A pool of 24 barcoded libraries (12 S-sEV and 12 L-sEV samples) was prepared and sequenced at the Functional Genomic Center Zurich (https://fgcz.ch) on an SP flow cell using an Illumina^®^ NovaSeq 6000 instrument (Illumina, Inc., San Diego, CA, USA). Paired-end sequencing was performed with 92 bp for the first read (cDNA insertion) and 8 bp for the second read (UMI sequence for PCR duplicate removal).

#### RNA-seq data analysis

Data analysis was performed in a locally installed version of Galaxy (https://usegalaxy.org) [25]. Sequencing reads were processed using Cutadapt (Galaxy version 1.16.8) with the parameters –u 1 (trims the first base to 50), –a A{10} (trims all poly(A) tracks and subsequent bases in the read), –m 15 (removes reads shorter than 15 bases) and a quality cutoff of 28. The trimmed reads were mapped to the current porcine genome reference assembly (Sscrofa11.1) using HISAT2 (Galaxy version 2.1.0 + galaxy4). NuDUP mark/remove PCR duplicates based on molecular tags (Galaxy version 2.3.3) was used to remove PCR duplicates from BAM files prior to counting reads mapped to annotated porcine genome features using the featureCounts tool (Galaxy version 1.6.4 + galaxy1). The most recent NCBI GFF3 genome annotation file (GCF_000003025.6_Sscrofa11.1) was used. Reads corresponding to mature miRNAs were counted separately using the MiRDeep2 quantifier (Galaxy version 2.0.0) based on the porcine, bovine, and human miRNA sequences from miRBase (version 22.1). MicroRNAs that showed at least 1 count in at least 1 sample were identified as detected miRNAs. MicroRNAs that showed at least 10 counts in at least 3 samples from the same pool were selected for further differential expression analysis.

Read count data were analyzed in R software (https://www.r-project.org) using the BioConductor edgeR package [26]. False discovery rates (FDR) < 0.1% to FDR < 10% were used to select differentially expressed genes (DEGs), differentially abundant (DA) RNAs between S- and L-sEV samples. An FDR < 0.1% was considered for highly significant.

To confirm the MiRDeep2 results, considering the complications and limitations of miRNA annotation [27], and to identify piRNA sequences, an additional sequence data analysis was performed as described previously [28]. Collapse Sequences (Galaxy version 1.0.1) was used to obtain unique sequences and corresponding counts for each sample. The sequence and count information for each sample was merged to create a read count table with the unique sequences as the identifier column. To remove sequences with negligible counts, filtering was performed with Filter countable by CPM cutoff (Galaxy version 1.2) (CPM cutoff 10, sample cutoff 3). This resulted in a total of 13,007 unique sequences, which were compared with NCBI BLAST + blastn (Galaxy version 2.10.1 + galaxy1) (blastn-short) against a variety of non-coding and coding sequence collections, including porcine, bovine, equine, mouse, and human miRNA sequences (mature and stem-loop sequences, miRBase version 22. 1), RFAM 14.7 sequences, porcine and human NCBI Refseq RNA sequences, porcine Ensembl 108 transcript sequences and porcine P-element-induced wimpy testes (PIWI)-interacting RNAs (piRNAs) from piRBase release 2.0 (http://bigdata.ibp.ac.cn/piRBase) [29].

#### Data mining and bioinformatics analysis of RNA EVs cargo

Gene symbols and Entrez Gene IDs (porcine) were mapped for all transcripts using custom bioinformatics tools integrated into a local Galaxy installation. Clustering analysis was performed using the Multiple Experiment Viewer tool (MeV v.4.8.1, https://sourceforge.net/projects/mev-tm4/) to generate HCL and SOTA expression plots [30]. Functional annotation analysis was performed using the online tool Metascape (www.metascape.org) [31]. MicroRNA target analysis was performed using MIENTURNET web tool (miRTarBase database) (http://userver.bio.uniromal1.it/apps/mienturnet [32] and DIANA tools MirPath v.3 (https://dianalab.e-ce.uth.gr/html/mirpathv3/index.php?r=mirpath) [33]. The Database for Annotation, Visualization, and Integrated Discovery (DAVID, https://david.ncifcrf.gov/) was used to identify enriched functional terms for miRNA target genes [34].

#### Data availability

RNA-Seq data have been deposited in the NCBI Sequence Read Archive (SRA) under the BioProject accession number PRJNA928243 (http://www.ncbi.nlm.nih.gov/bioproject/928243).

### Statistical analysis

Data of the sEV characterization parameters were analyzed using the GraphPad Prism 10.1.0 statistical package (GraphPad Software, Inc., La Jolla, CA, USA; https://www.graphpad.com/). Normality of the data distribution was analyzed using the Shapiro-Wilk test and differences between the two sEV size subsets (S-sEVs vs. L-sEVs) were analyzed using the t-test for unpaired data or the Mann-Whitney test. Differences were considered statistically significant at *P* < 0.05.

## Results

### Characterization of S-sEVs and L-sEVs

**Fig. 1:**
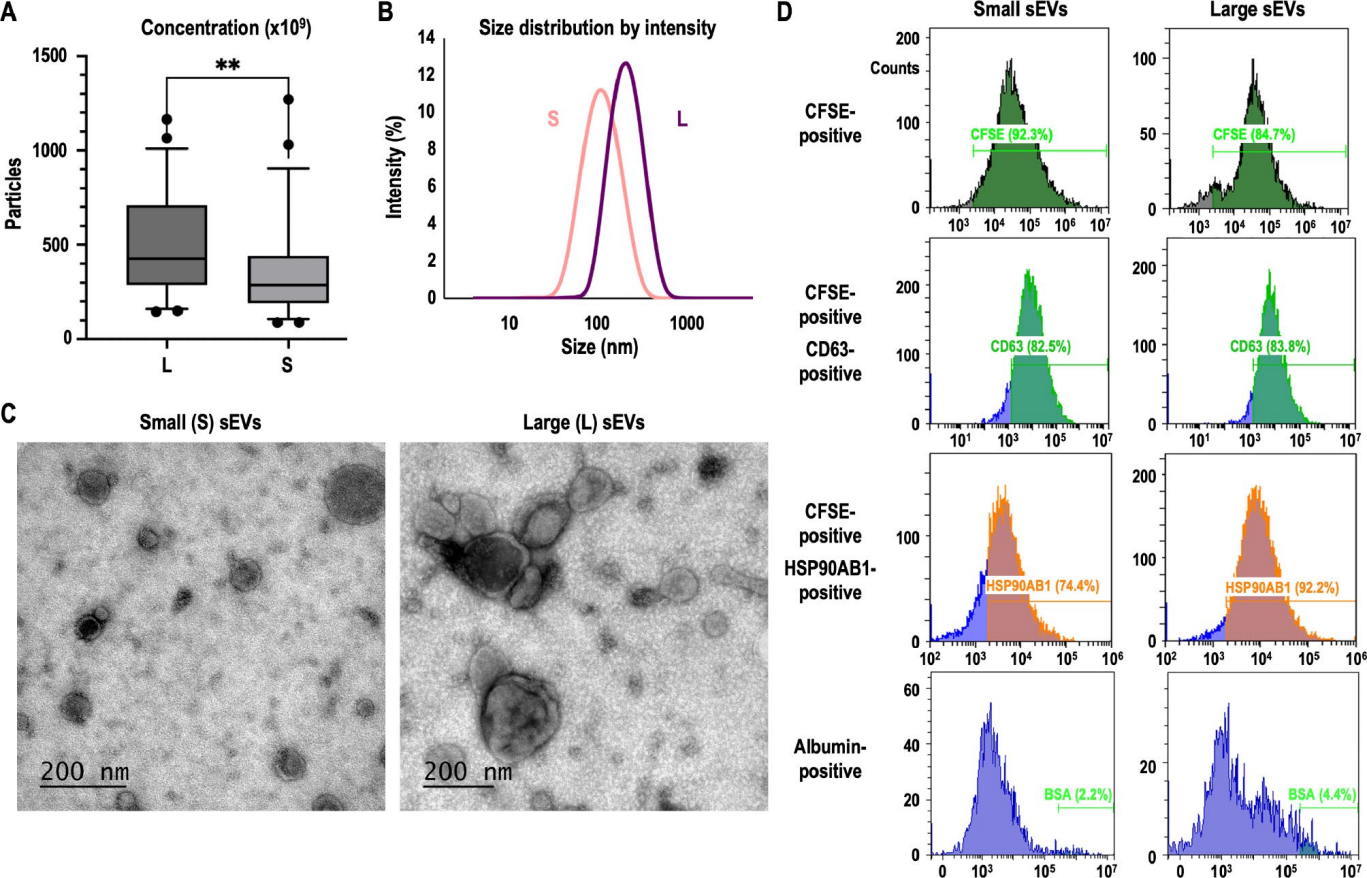
Characterization of porcine small (S) and large (L) seminal extracellular vesicles (sEV) samples. (**A**) Particle concentration; (**B**) particle size distribution; (**C**) sEV morphology; and (**D**) Flow cytometry measurements of percentages of sEVs (CFSE-positive particles), sEV positive to protein markers CD63 and HSP90β, and of non-vesicular extracellular particles (positive to albumin)

Total protein concentration (mean ± SD) was higher (*P* < 0.001) in S-sEV samples (269.2 ± 152.5 µg/mL) than in L-sEV samples (68.5 ± 37.8 µg/mL). Particle concentration (mean ± SD) was higher (*P* < 0.01) in L-sEV samples (494.8 × 10^9^ ± 272.5 × 10^9^ particles/mL) than in S-sEV samples (348.8 × 10^9^ ± 245.3 × 10^9^ particles/mL) (Fig. 1A). The particle size distribution of the S-sEV samples (75 and 160 nm for the 25% and 75% percentiles) was different (*P* < 0.001) from that of the L-sEV samples (150 and 310 nm for the 25% and 75% percentiles) (Fig. 1B). The morphology of the sEVs also showed clear differences between the S-sEV and L-sEV samples. While the S-sEV samples contained mainly spherical sEVs, the L-sEV samples had sEVs with a more heterogeneous morphology (Fig. 1C). Flow cytometry analyses revealed differences (*P* < 0.001) in the percentage of CSFE-positive particles between S-sEV (mean ± SD: 84.1 ± 2.5%) and L-sEV (77.3 ± 3.7%) samples. The percentage (mean ± SD) of CSFE-positive particles expressing CD63 was similar in S-sEV and L-sEV samples (ranging from 76.9 to 91.0%), whereas that of HSP90β was higher (*P* < 0.05) in L-sEV (87.2 ± 6.6%) than in S-sEV (80.6 ± 7.4%) samples (Fig. 1D). Flow cytometry analysis also showed that both L-sEV and S-sEV samples had low percentages of non-vesicular extracellular particles (albumin-positive particles below 6% in all sEV samples), which would indicate a high degree of purity of sEVs (Fig. 1D). Characterization data for each of the 24 sEV samples are provided in Additional file 1.

### RNA content of sEVs

RNA concentrations were lower (*P* < 0001) in the S-sEVs samples (ranging from 0.14 ng/µL to 0.36 ng/µL) than in the L-sEVs samples (ranging from 0.5 ng/µL to 1.73 ng/µL) (Additional file 2 and Additional file 3). Both S-sEV and L-sEV samples had a similar RNA profile in all samples, characterized by a single peak between 20 and 150 nt.

**Fig. 2:**
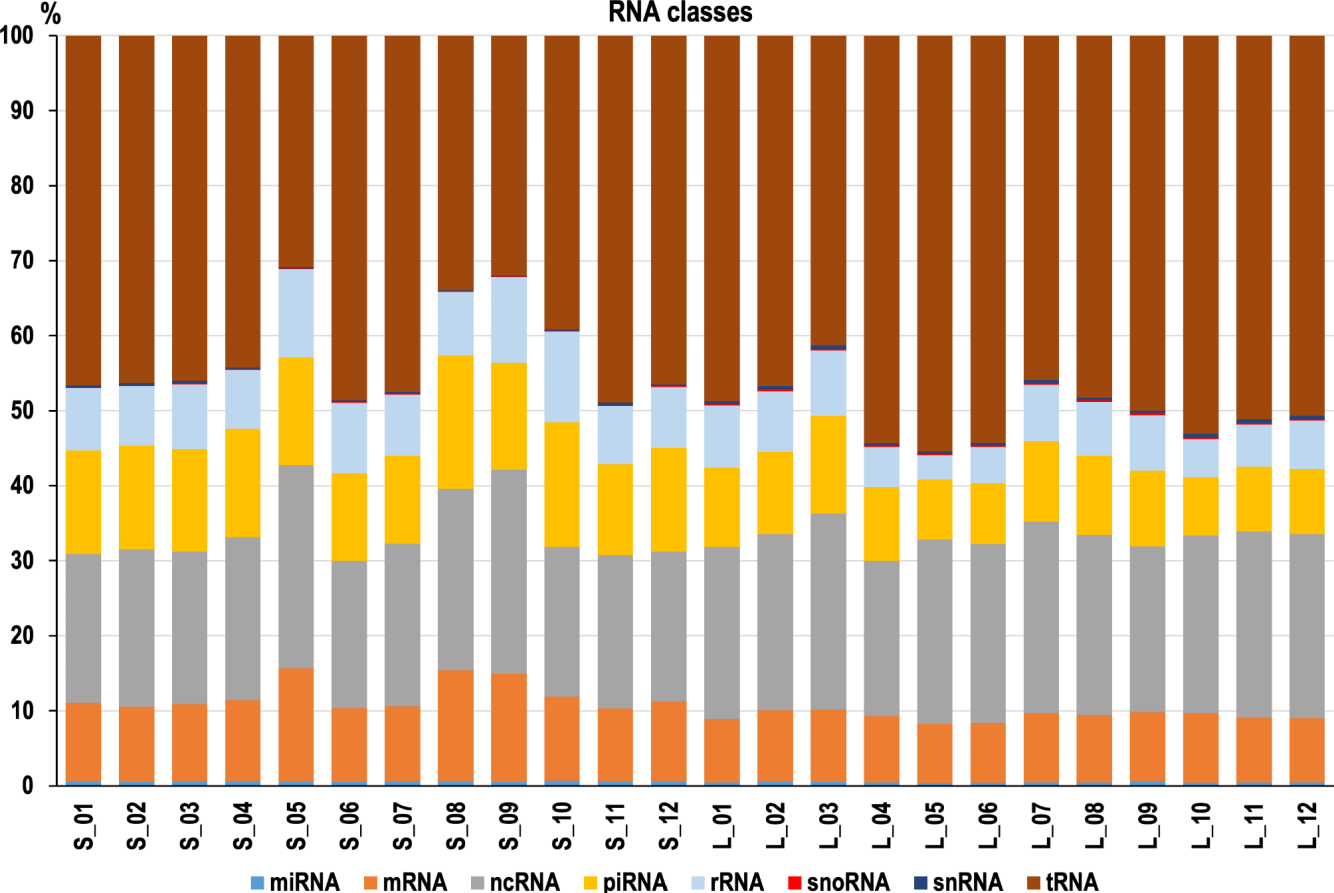
Bar graph showing the distribution of RNA classes found in porcine seminal plasma extracellular vesicles (sEVs). Each column shows the percentage of reads assigned to each RNA class in each of the 12 small (S) and large (L) sEV samples analyzed. Transfer RNA (tRNA); microRNA (miRNA); piwi-interacting RNA (piRNA); protein-coding RNA (mRNA); ribosomal RNA (rRNA), small nuclear RNA (snRNA), small nucleolar RNA (snoRNA), and other non-coding RNA (ncRNA)

RNA sequencing analysis identified several RNA classes among the sequences derived from the S-sEV and L-sEV samples, namely protein-coding RNAs (messenguer RNA (mRNAs)) and several non-coding RNA species, specifically ribosomal RNAs (rRNAs), miRNAs, piRNAs, other non-coding RNAs (ncRNAs), transfer RNAs (tRNAs), small nucleolar RNAs (snoRNAs) and spliceosomal U small nuclear RNAs (snRNAs). In descending order, tRNAs, ncRNAs, piRNAs, mRNAs, and rRNAs were the most abundant RNA classes in terms of the percentage of reads (Fig. 2). Raw data for the “all RNAs” identified in each of 12 samples of S-sEVs and 12 samples of L-sEVs are shown in Additional file 4 (Table S1). The mean percentage of read counts of each of the identified RNA classes differed (*P* < 0.05) between S-sEV and L-sEV samples (Table 1 and Additional file 4: Table S2).

**Table 1:** Comparison of the percentage of read counts for different classes of RNAs between small (S) and large (L) extracellular vesicles isolated from porcine seminal plasma. Transfer RNA (tRNA), other non-coding RNA (ncRNA), Piwi-interacting RNA (piRNA), protein-coding RNA (mRNA), ribosomal RNA (rRNA), microRNA (miRNA), small nuclear RNA (snRNA) and small nucleolar RNA (snoRNA)

RNA class	S	L	Fold change S vs. L	*P*-value
tRNA	42.55	49.98	0.85	0.0042
other ncRNA	21.91	23.85	0.92	0.046
piRNA	14.01	9.76	1.44	< 0.00001
mRNA	11.44	8.87	1.29	0.0011
rRNA	9.16	6.47	1.42	< 0.001
miRNA	0.59	0.45	1.31	< 0.0001
snRNA	0.22	0.43	0.52	< 0.00001
snoRNA	0.12	0.20	0.59	0.00015

According to the filter criteria defined in the RNA-seq data analysis, a total of 369 RNAs (“all RNAs”) were selected for the statistical analysis, including mRNAs, miRNA precursors, tRNAs, rRNAs, snoRNAs, snRNAs and other ncRNAs (Additional file 4: Table S3). Similarly, 40 miRNAs with at least 10 counts in three samples (Additional file 4: Table S4) and 326 identified piRNAs (Additional file 4: Table S5) were taken into acount for the statistical analysis.

### Difference between S- and L-sEVs in RNA cargo

**Fig. 3:**
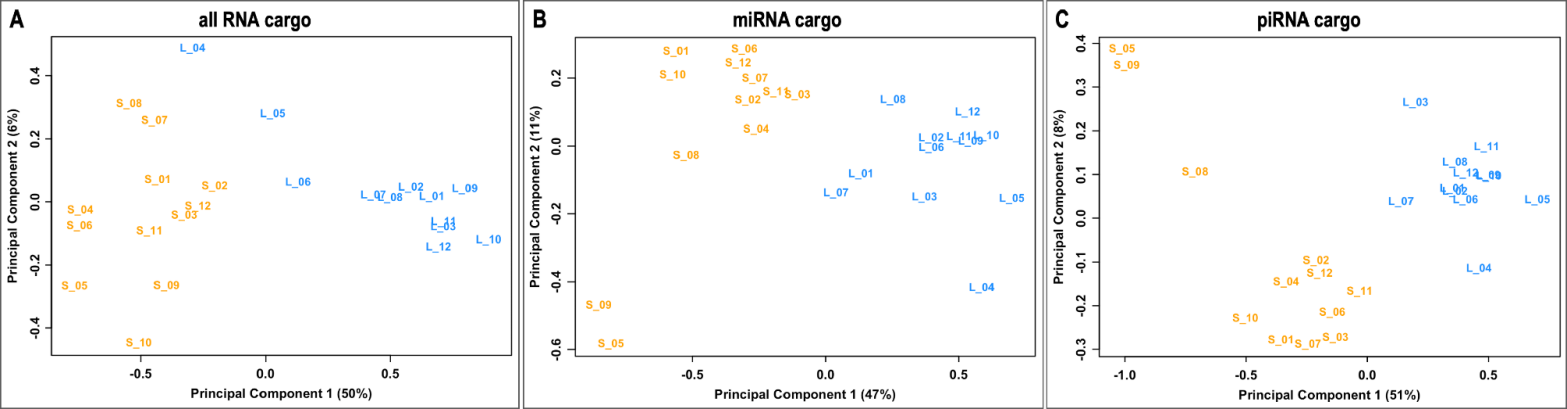
Two-dimensional principal component analysis showing cargo differences for (**A**) “all RNAs” (including ribosomal RNAs, other non-coding RNAs, transfer RNAs, small nucleolar RNAs and small nuclear RNAs), (**B**) microRNAs (miRNAs) and (**C**) PIWI-interacting RNAs (piRNAs) between small (S, *n* = 12) and large (L, *n* = 12) extracellular vesicle samples of porcine seminal plasma

**Fig. 4:**
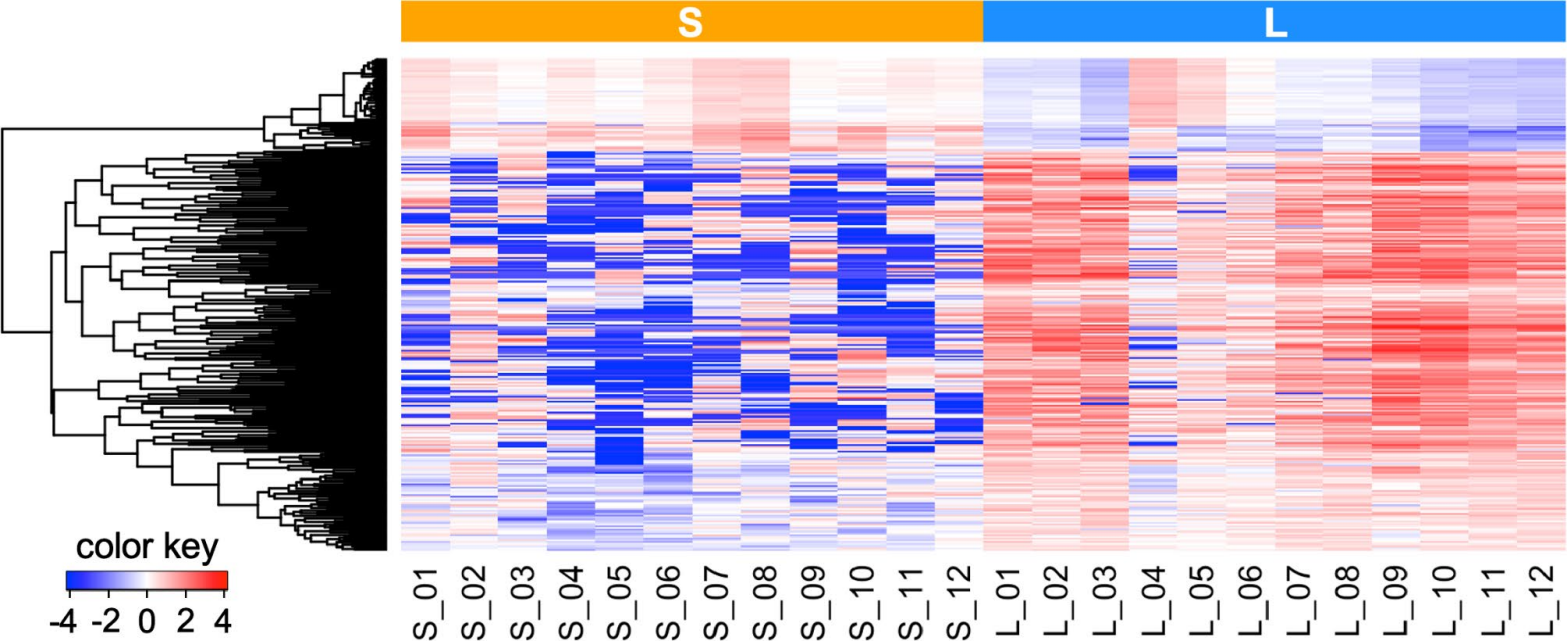
Dendrogram from an unsupervised hierarchical clustering analysis showing the differential cargo of 194 selected “all RNAs” with an FDR of < 0.1% between small (S, *n* = 12) and large (L, *n* = 12) extracellular vesicles (sEV) samples isolated from porcine seminal plasma. Rows show individual RNAs and columns show individual samples of sEVs. Data are mean-centered expression values (log_2_ counts per million samples - mean of log_2_ counts per million of all samples). The color scale ranges from − 4 (blue, below the mean) to 4 (red, above the mean). “All RNAs” includes protein-coding RNAs, ribosomal RNAs, other non-coding RNAs, transfer RNAs, small nucleolar RNAs, and small nuclear RNAs

Analysis of the RNA cargo using principal component analysis showed a clear separation between S-sEV and L-sEV samples for “all RNAs”, miRNAs, and piRNAs (Fig. 3). Of the 369 “all RNAs”, 277 (75.1%) and 194 (52.6%) were DA between S-sEV and L-sEV samples with an FDR < 1% and < 0.1%, respectively (Additional file 4: Table S6). Hierarchical clustering analysis of the 194 DA “all RNAs” with an FDR of < 0.1% revealed a distinct pattern between S-sEVs and L-sEVs (Fig. 4). A total of 39 and 102 “all RNAs” were found to be of higher and lower abundance, respectively, in all S-sEV samples compared to the L-sEV samples. Among them, 24 of the 45 tRNAs identified were DA between S-sEV and L-sEV samples (Additional file 4: Table S6). Most of the mRNAs were more abundant in L-sEVs, whereas most of the tRNAs were more abundant in S-sEVs.

**Fig. 5:**
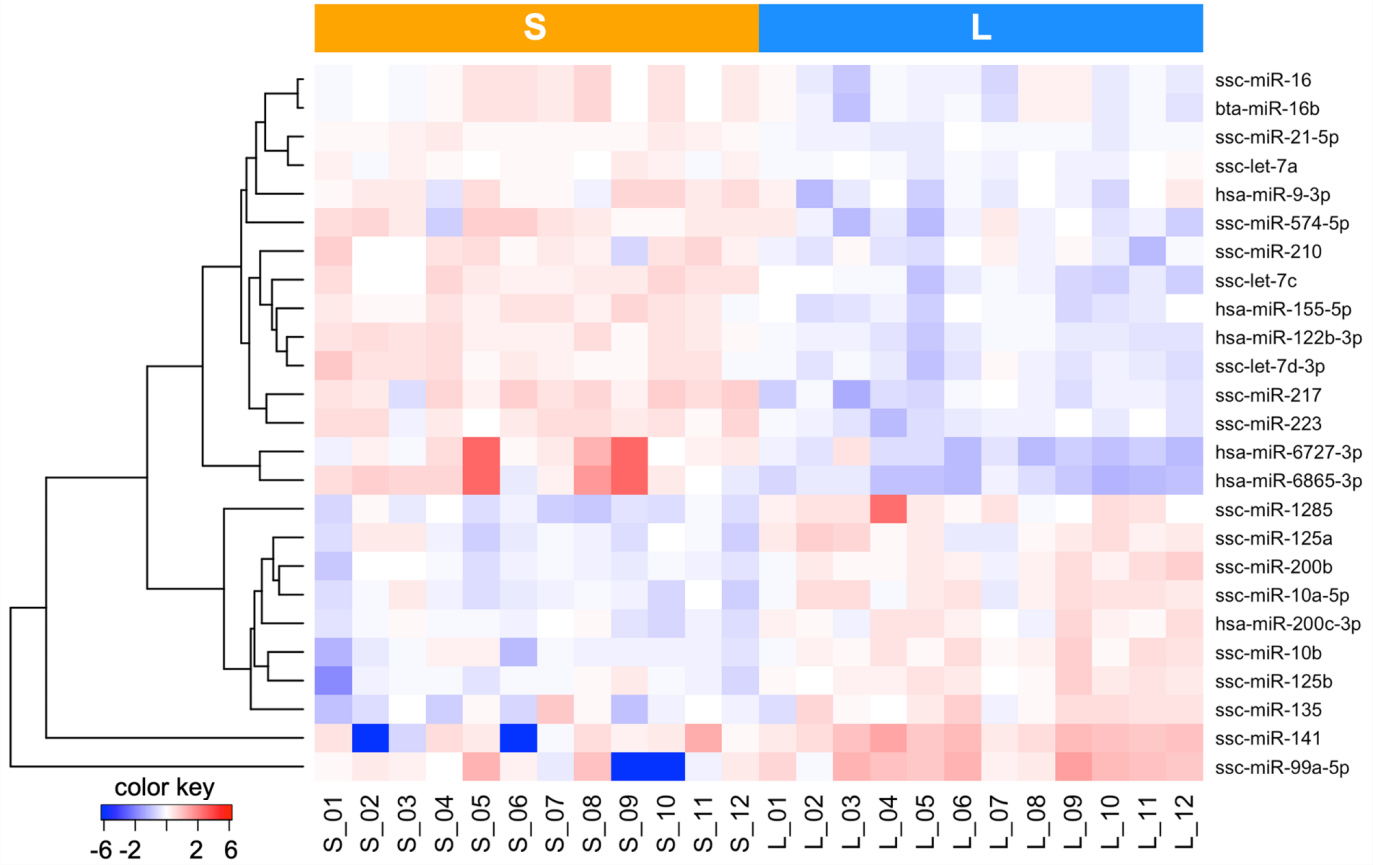
Dendrogram from an unsupervised hierarchical clustering analysis showing the differential loading of 25 selected microRNAs (miRNAs) with an FDR of < 0.1% between small (S, *n* = 12) and large (L, *n* = 12) extracellular vesicle (sEV) samples isolated from porcine seminal plasma. Rows show individual miRNAs, and columns show individual samples of sEVs. Data are mean-centered expression values (log_2_ counts per million samples - mean of log_2_ counts per million of all samples). The color scale ranges from − 6 (blue, below the mean) to 6 (red, above the mean)

Of the 40 selected miRNAs, 30 (75%) and 25 (62.5%) were DA between S-sEV and L-sEV samples with an FDR < 1% and < 0.1%, respectively (Additional file 4: Table S7). Hierarchical cluster analysis of the 25 DA miRNAs with an FDR < 0.1%, revealed 15 and 10 miRNAs with higher and lower abundance in S-sEV samples compared to L-sEV samples (Fig. 5). Further miRNA annotations based on BLAST comparisons with different sequence databases revealed that some of the sequences annotated in miRBase as miRNAs might be derived from other ncRNAs. In particular, the sequences of miR-1285 matched with *RN7SL*, a signal recognition particle RNA; the sequences of miR-2478 and miR-4286 were consistent with piRNAs, and those of miR-2887 were consistent with 28 S rRNA (Additional file 4: Table S8).

**Fig. 6:**
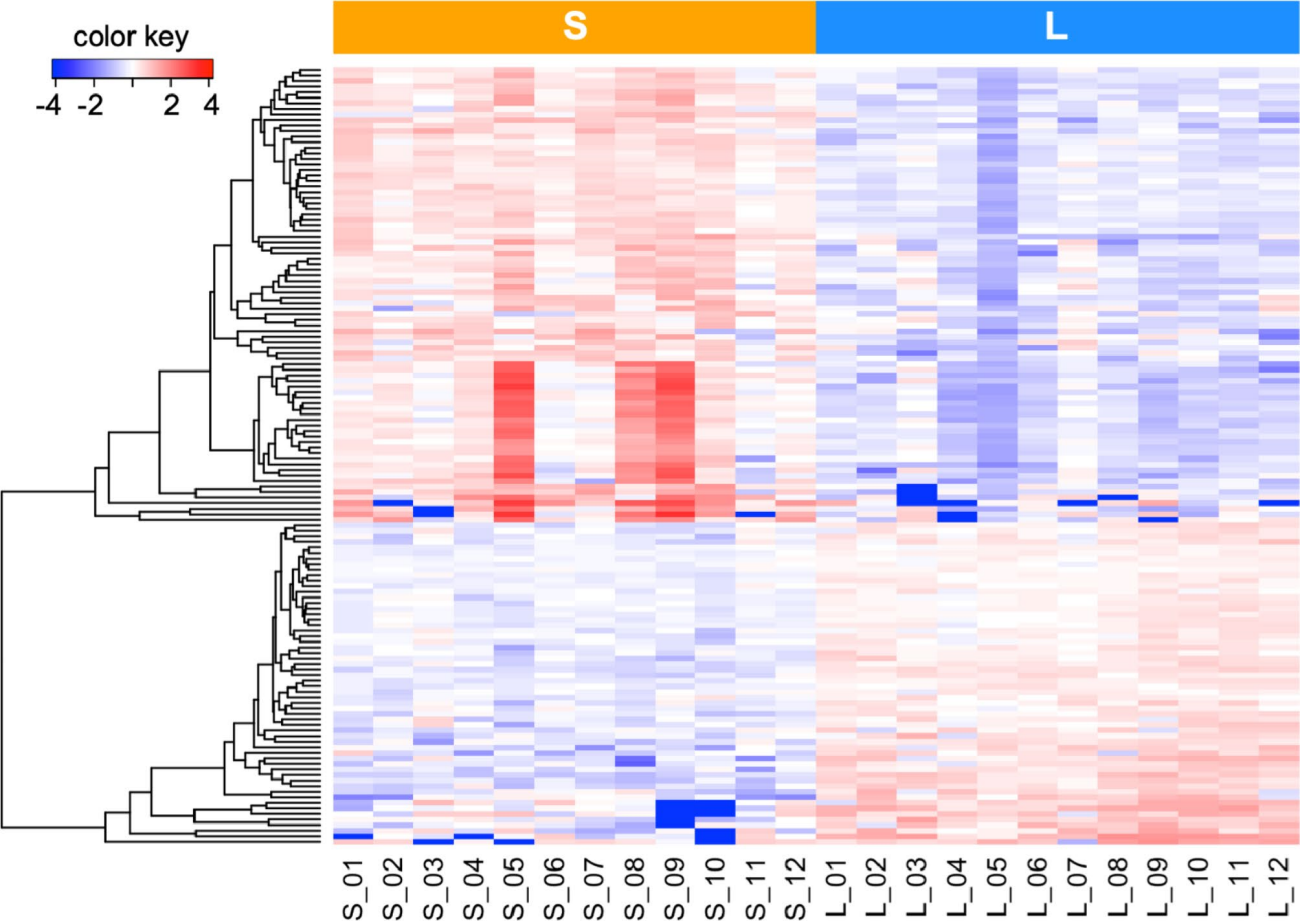
Dendrogram from an unsupervised hierarchical clustering analysis (with Pearson correlation coefficient by MeV) showing the differential loading of 140 selected piwi-interacting RNAs (piRNAs) with an FDR of < 0.1% between small (S, *n* = 12) and large (L, *n* = 12) extracellular vesicle (sEV) samples isolated from porcine seminal plasma. Rows show individual piRNAs, and columns show individual samples of sEVs. Data are mean-centered expression values (log_2_ counts per million samples - mean of log_2_ counts per million of all samples). The color scale ranges from − 4 (blue, below the mean) to 4 (red, above the mean)

Among the 326 identified piRNAs, 187 (57.4%) and 140 (42.9%) were DA between S-sEV samples and L-sEV samples with an FDR < 1% and < 0.1%, respectively (Additional file 4: Table S5). Hierarchical cluster analysis of the 140 DA piRNAs with an FDR of < 0.1% revealed a distinct pattern between S-sEVs and L-sEVs (Fig. 6). Approximately half of these DA piRNAs were more abundant in the S-sEV samples and the other half were more abundant in the L-sEV samples. The most DA piRNAs with a log_2_ fold change of ≥ 1, were more abundant in S-sEV samples (n:79) than in L-sEV samples (n:25).

### Functional analysis for differentially abundant RNAs

**Fig. 7:**
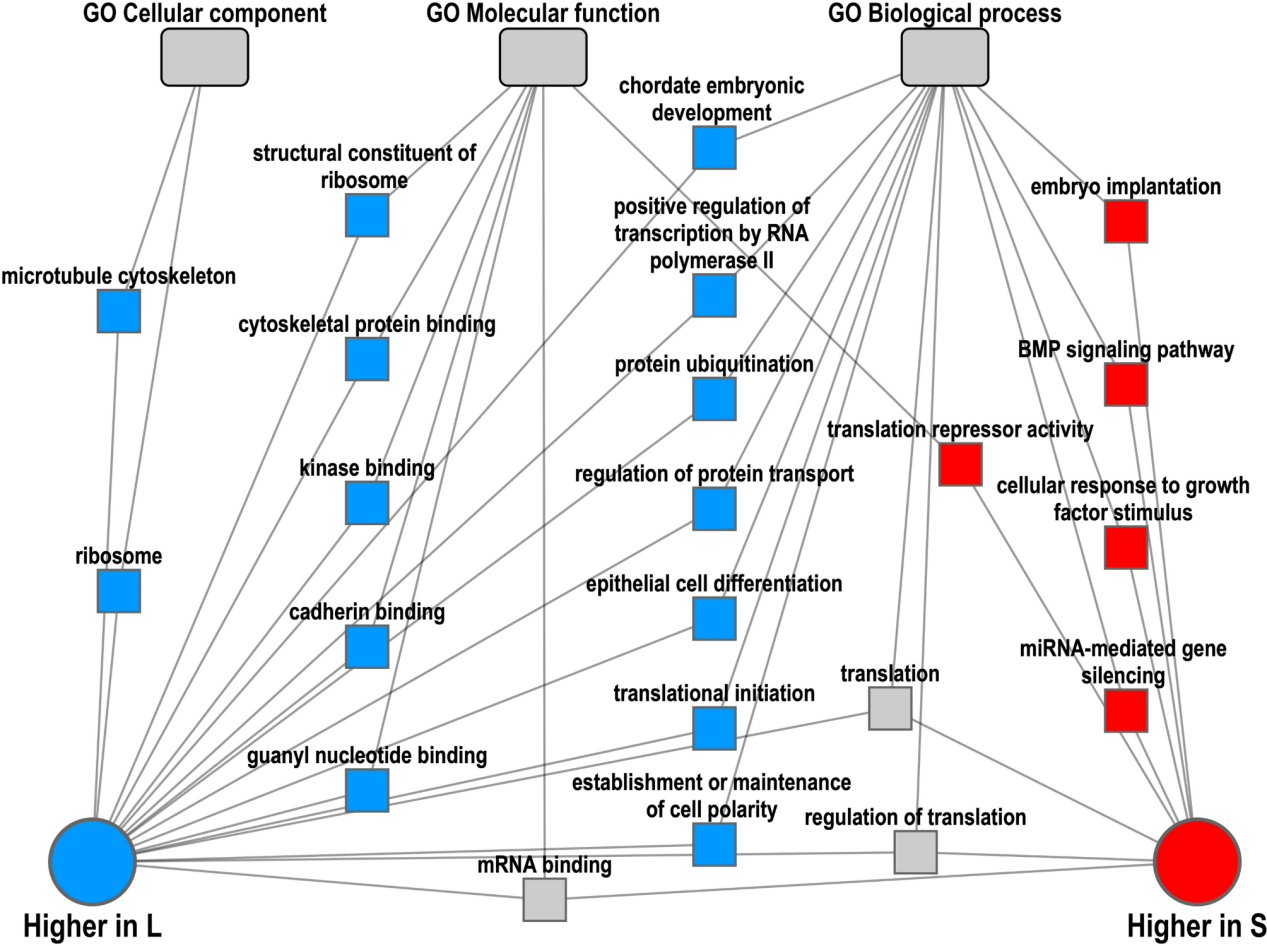
Network of overrepresented functional terms for “all RNAs” differentially abundant in small (S) and large (L) porcine seminal plasma extracellular vesicles (sEVs). Functional annotation enrichment analysis was performed using ToppCluster (https://toppcluster.cchmc.org/)

To perform functional annotation enrichment analysis of the DA “all RNAs”, ToppCluster (https://toppcluster.cchmc.org/) [35] was used to generate a network of genes and overrepresented functional categories. Analysis of “all RNAs” more abundant in L-sEVs revealed enriched functional categories related to ribosome, translation, cytoskeleton, protein transport, epithelial cell differentiation and chordate embryonic development (Fig. 7 and Additional file 5). Analysis of “all RNAs” more abundant in S-sEVs revealed enriched functional categories related to bone morphogenetic protein (BMP) signaling pathway, translation repressor activity, miRNA-mediated gene silencing and embryonic implantation (Fig. 7).

**Fig. 8:**
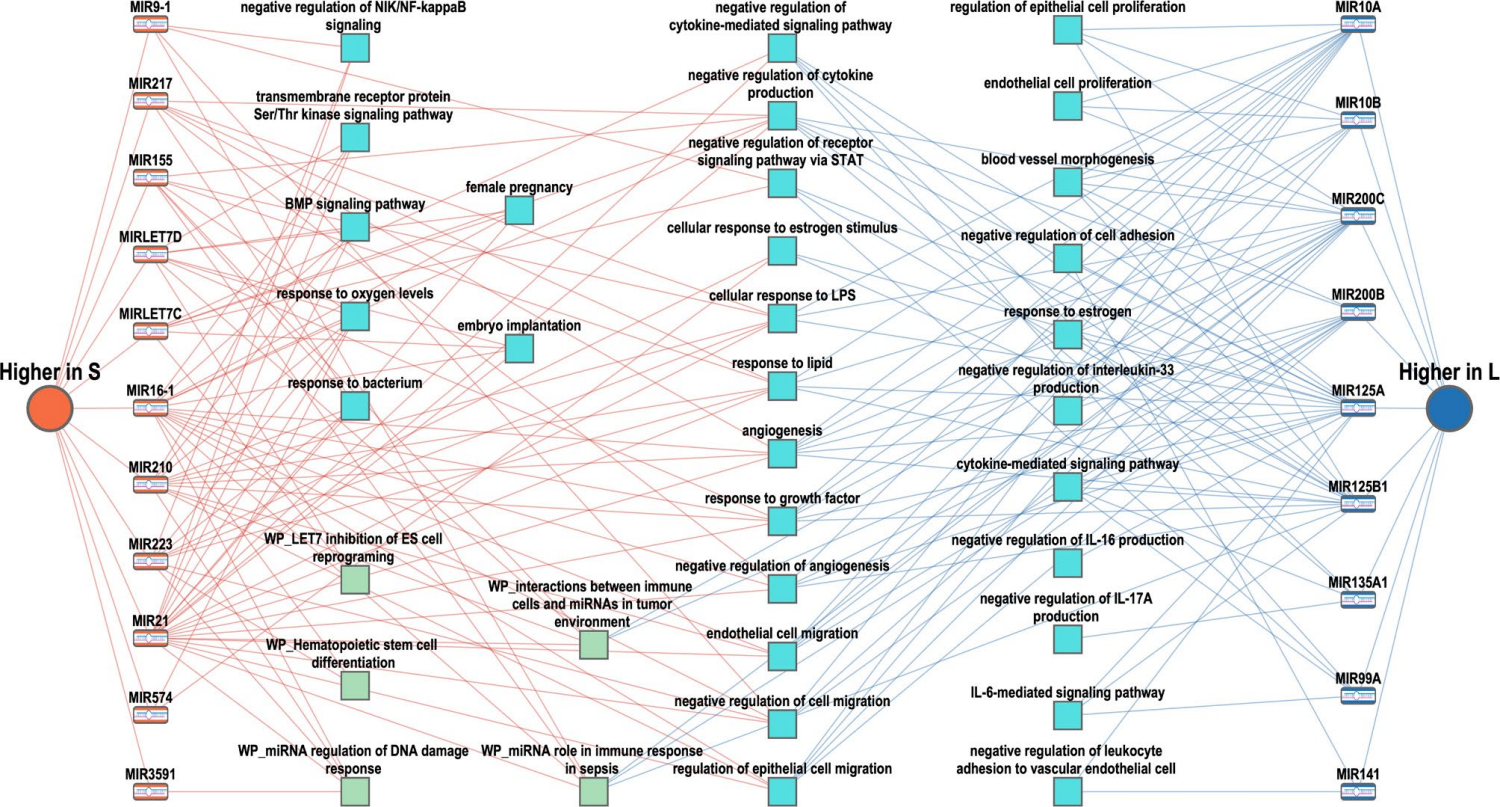
Network of overrepresented functional terms for microRNAs differentially abundant in small (S) and large (L) porcine seminal plasma extracellular vesicles (sEVs). Functional annotation enrichment analysis was performed using ToppCluster (https://toppcluster.cchmc.org/)

For the DA miRNAs, the network of miRNAs and overrepresented functional categories generated by ToppCluster showed that DA miRNAs were enriched for functional categories related to immune response, cytokine signaling, angiogenesis, response to a variety of factors, and pregnancy-associated categories (Fig. 8 and additional file 4 (Table S9)). The more abundant miRNAs in S-sEVs were specifically involved in functional categories such as BMP signaling pathway, response to oxygen levels, DNA damage response, response to bacteria, female pregnancy, and embryo implantation. The more abundant miRNAs in L-sEVs were specifically related to categories such as negative regulation of interleukin production (IL6, IL16, IL17A, and IL33), blood vessel morphogenesis, and endothelial and epithelial cell proliferation.

## Discussion

The present study demonstrated that the RNA load of two different-sized subsets of EVs isolated from porcine SP exhibited quantitative differences with potential functional implications. This finding is novel for body fluid-derived EVs and is consistent with the results of Paloczi et al. [36] who observed differences in RNA content in subsets of EVs of different sizes isolated from the TIB202 cell line. The differences in RNA content between large and small sEVs are also consistent with the previously observed differences in protein content and lipid composition between the two sEV subsets [21, 37]. The different cellular source would explain the differences in composition between large and small sEVs [19]. Furthermore, the differences in composition should be key to explaining the different functional roles of small and large sEVs [20]. In some cancers, such as melanoma, differences in RNA cargo between subsets of EVs have helped to understand disease initiation and progression [38].

### RNA content of sEVs

This study provides a landscape of the total RNA cargo of porcine sEVs, which was made possible for the first time by RNA-seq libraries containing both short and long RNA molecules. Accordingly, the presence of mRNAs, rRNAs, ncRNAs, tRNAs, snoRNAs and snRNAs was identified in the two subsets of sEVs, in addition to the more commonly identified miRNAs and piRNAs. Studies on the RNA load of porcine seminal EVs have been published in the last few years [15, 39–42]. These studies have mainly focused on miRNAs. However, some of them have also reported the presence of other RNAs, although not using RNA-seq libraries. For example, Xu et al. [41] also identified piRNAs, mRNA fragments, and small RNAs derived from tRNAs using a small RNA-seq library approach. Ding et al. [40] performed total RNA-seq for long RNAs in addition to small RNA-seq and identified different classes of short and long ncRNAs. Taken together, these studies and our own suggest that RNAs, other than the commonly studied miRNAs, may be involved in the functional performances that are attributed to sEVs.

The sequences assigned to tRNAs, could be derived from tRNA halves (tsRNAs) that are potentially involved in the regulation of gene expression, development, and differentiation [43]. Xu et al. [41] previously reported their presence in porcine sEVs, although at a lower proportion. The tsRNAs have been identified in gametes and embryos and would be involved in gamete maturation, fertilization, and early embryonic development [44–46]. Spermatozoa are particularly enriched in tsRNAs that would be of sEV origin [47, 48]. These findings support the role of sEVs in providing RNAs to spermatozoa and suggested that tsRNAs would be among the most transferred. Some tsRNAs identified in sperm have been shown to play important roles in epigenetic inheritance [45, 49, 50]. These results call for further studies of sEV tsRNAs, as they may be relevant to the paternal epigenetic information transferred from the sperm to the oocyte at fertilization [49]. Interestingly, some of the tsRNAs identified in the present study have been proposed as candidate biomarkers for human male infertility disorders [51].

Other ncRNAs were also abundant in both sEV subsets. In fact, they were the second most abundant in percentage terms after tsRNAs. Their high presence in EVs is consistent with previous studies [52]. They have also been identified in bovine oviductal EVs [53] and they could include YRNA-derived fragments [54]. To our knowledge, they have not been associated with reproductive (dys) function.

The piRNAs are small RNAs that are widely expressed in the male reproductive tract, including SP [47]. A large number of them were identified in the sEVs analyzed in the present study, which is in agreement with previous studies in human sEVs [13, 55] and also in porcine sEVs [41]. Unfortunately, the biogenesis and the role of EV piRNAs are still not well understood [56]. The piRNAs play an essential role in the silencing of transposable elements and thus in the maintenance of genome stability [57]. Therefore, they would be essential for maintain genome integrity during spermatogenesis and early embryo development [58, 59]. Accordingly, defective piRNA signaling results in impaired spermatogenesis and infertility [60]. The piRNAs would also be required for epididymal sperm maturation [61]. Changes in the expression of some seminal piRNAs have been associated with azoospermia, oligospermia and asthenozoospermia [44, 51]. Thus, some seminal piRNAs have been explored as putative biomarkers of semen quality [51].

Ten percent of the RNAs identified in sEVs were mRNAs. Transcripts introduced into cells by EVs are rapidly degraded without being translated into proteins [62]. However, mRNAs loaded into EVs are stable molecules that can be transferred to recipient cells, where they would be transcribed into functional proteins, modifying the functional response of the target cells [63, 64]. Thus, it has been shown that after EV uptake, some mRNAs contained in oviductal EVs are upregulated in embryos [65, 66]. These mRNAs have been associated with improvements in embryo development [65, 66]. The EVs deliver their cargo to sperm [67]. Seminal EVs would be involved in the regulation of essential sperm functions such as motility and capacitation [68, 69], and sEV mRNAs could be intermediates in this sEV regulatory role. The mRNAs identified in the sEV isolated in the present study encoding proteins involved in sperm functionality would support this hypothesis. In particular, the mRNAs encoding the spermadhesins PSPI, PSPII, AWN, AQN-1 and a protein of the sperm binding protein family have been identified in the sEVs isolated in the present study. All these proteins are involved in regulating sperm motility, capacitation, acrosomal reaction and binding to the pellucid zone [70–72] .

The rRNAs represented 5–10% of the RNAs identified in the sEVs. Unfortunately, rRNA was not analyzed because rRNA depletion was performed during library preparation to increase the detection of other RNA types. Since the levels of rRNA fragments were not known prior to removal, the removal of rRNA may have biased the observed differences. However, since the EV RNA cargo is mainly characterized by small fragments, the removal of rRNA should be considered beneficial, as it allows the identification of a larger number of other small ncRNAs [73].

The miRNAs were present in both subsets of sEVs, but they represented less than 1% of the total number of RNAs counted. Nevertheless, they are among the most studied sEV-loaded molecules. The sEVs miRNAs can be transferred to the spermatozoa [51] and they play key roles in spermatogenesis, sperm maturation, fertilization, and embryo development. Moreover, profile changes in some specific miRNAs have been associated with male fertility disorders [47, 51]. Previous studies have identified a large number of miRNAs in porcine sEVs [15, 39–42]. Compared to these studies, our study is the one that identified the highest number of miRNAs in porcine sEVs, with a total of 540 miRNAs identified with at least one read count. Beyond the total number of miRNAs, it was not possible to verify how many miRNAs match between studies. This is because most of the studies mentioned above do not provide lists of identified miRNAs. An exception is the study by Zhang et al. [42], which identified 162 miRNAs, 123 of which matched with the miRNAs identified in the present study. Xu et al. [41] and Dlamini et al. [15] just listed the most highly expressed miRNAs, and all of them were also identified in the present study (see Additional file 6). This high level of concordance increases confidence in the results of this study. Some of the miRNAs identified in the sEVs and selected according to the criteria outlined in the methodology have been reported in porcine spermatozoa and were either related to spermatogenesis [74] or even postulated as fertility biomarkers for boars used in AI programs [75].

Small nucleolar RNAs and snRNAs were present in both subsets of sEVs, but at relatively low levels. Together they represent less than 1% of the total RNAs counted. Their low abundance in sEVs may be due to their nuclear nature [76]. Therefore, they are not easily transferred to EVs from EV-secreting cells. Small nucleolar RNAs have gained scientific interest in recent years and are recognized by their surprising functional versatility, which would give them a broad impact on cellular functionality [77]. Unfortunately, most of the snoRNAs are still annotated as orphans and their cellular functionality is not known. Small nuclear RNAs would play an essential role in the functional and regulatory activities of cells as they are directly involved in RNA modification/processing, including pre-mRNA splicing [76]. To the best of our knowledge, the involvement of snoRNAs and snRNAs in reproductive functions is still lacking [77].

### Differential RNA cargo between S-sEVs and L-sEVs

Quantitative differences in RNA content between S- and L-sEVs were found for tsRNAs, piRNAs, mRNAs and miRNAs. A large number of tsRNAs were shown to be DA between the two subsets of sEVs. Most of them were found to be more abundant in S-sEVs, including some of those enriched in spermatozoa and involved in epigenetic inherence [45, 49]. Similarly, more than half of piRNAs differed in abundance between the two sEV subsets. Small sEVs had more overabundant piRNAs than L-sEVs when considering the most DA piRNAs (log_2_ fold-change ≥ 1.25). Thus, it could be hypothesized that S-sEVs would be more relevant than L-sEVs in terms of functional performance associated with piRNAs. Unfortunately, due to the current lack of knowledge about the role of EV piRNAs, no solid conclusions can be drawn from the results of the present study. Most of the DA mRNAs were found overabundant in L-sEVs. Small sEV samples would contain more exosomes and L-sEV samples would contain more ectosomes or microvesicles [18] . Exosomes and ectosomes differ in the biogenesis pathway, and the mRNA content would be better expressed in ectosomes [78]. Therefore, in terms of the role of EV mRNAs, L-sEVs would have a higher functional capacity than S-sEVs. Unfortunately, the role of sEV mRNAs in male fertility is not yet known. However, the possible functional role of some of them could be hypothesized. For instance, for sperm-associated antigen 9 (SPAG9), which was found to be more abundant in L-sEVs. SPAG9 is an acrosome-specific sperm protein that translocate to the equatorial region during the acrosomal reaction and is therefore strongly associated with sperm oocyte binding function [79]. Other mRNAs that were found to be more abundant in L-sEVs than S-sEVs include AMOT, SMIM14, PAFAH1B1, FN1, TEAD1, CTNNB1, DLX1, and KRT8. Many of these mRNAs have been described as important for early embryo development [80–82]. As occurs in early embryos that increase mRNA following oviductal EV uptake, one might speculate that the above sEV mRNAs would be transferred to the sperm following sEV uptake and transferred from the sperm to the oocyte at the time of fertilization.

Many miRNAs were also found to be DA between S-sEVs and L-sEVs, and some of them were reported to be related to sperm functionality and performance, and male fertility. Among the miRNAs more abundant in S-sEVs, the seminal miR-155-5p was reported to be positively related with sperm motility in pigs [40]. Interestingly, it was also found at high levels in cumulus cells of porcine oocytes with good in vitro maturation and cleavage rate [83]. However, other miRNAs that were more abundant in S-sEVs had negative effects. For example, miRNA let-7c was upregulated in SP of men with azoospermia and higher seminal bisphenol A levels [84]; and miRNA let-7d was upregulated in porcine spermatozoa with morphological abnormalities or low motility [85]. Among the miRNAs more abundant in L-sEVs, sperm levels of miR-99a-5p were positively associated with sperm cryopreservation in bulls [86]. Similarly, sperm miR-10a-5p levels were positively associated with fertility of sex-sorted X-bearing sperm in cattle [87] and sperm cryopreservation success in pigs [88, 89]. Also, miR-10a-5p was found at high levels in low motile bull spermatozoa [86]. Similarly, miR-1285 was found upregulated in the spermatozoa of highly fertile boars [75]. However, porcine miR-1285 annotated in miRBase is probably not a miRNA but is derived from RN7SL, a structural RNA component of the signal recognition particle. The miRNAs miR-10a-5p, miR-10b and miR-135, also found at high levels in L-sEVs, were found at low levels in SP of infertile men [90]. In contrast, miR-141 levels in SP and sperm were found to be negatively correlated with ejaculate sperm concentration in men [91]. Taken together, these findings support what was already proposed, that many of the sEV miRNAs may be involved in sperm functionality, including fertility. However, and as a novelty, they also highlight that the functional profile of sEVs is not unidimensional, at least as far as miRNAs are concerned, as S-sEVs and L-sEVs show differences in the cargo of miRNAs potentially related to sperm functionality and fertility.

## Conclusions

The S- and L-sEVs isolated from porcine SP show quantitative differences in RNA content. Such differences would suggest that the two sEV subsets may have different functional roles. This hypothesis is supported by the recognized reproductive roles, including fertility, of some of the miRNAs, tsRNAs, mRNAs, and piRNAs that were found to be DA between S-sEVs and L-sEVs. The isolation of each of the EV subpopulations/subsets coexisting in SP is an open challenge. Its achievement will increase the current knowledge of the specific functional role of each sEV subpopulation. This will lead to the identification of fertility biomarkers in S-sEVs and L-sEVs and the potential use of specific sEV subpopulation/subset as media additives to improve the efficiency of assisted reproductive technologies.

## Electronic supplementary material

Below is the link to the electronic supplementary material.

**Additional file 1** (.xlsx). Raw data from the characterization of small (S-, *n* = 12) and large (L-, *n* = 12) extracellular vesicle (EV) samples isolated from porcine seminal plasma. (A) Size distribution (nm) and particle concentration (particles/mL) quantified by nanoparticle tracking analysis (NTA). (B) Presence of EV-specific protein and non-vesicular extracellular particle markers analyzed by flow cytometry (CytoFLEX S): (1) carboxyfluorescein succinimidyl ester (CSFE)-positive particles (EV, %), (2) CSFE-positive particles expressing CD63 (%), (3) CSFE-positive particles expressing HSP90β (%), and (4) albumin-positive particles (%)

**Additional file 2** (.ppt). **Figure S1**. RNA profiles and concentrations of the 12 samples of small extracellular vesicles isolated from porcine seminal plasma that were used for RNA sequencing

**Additional file 3** (.ppt). **Figure S2**. RNA profiles and concentrations of the 12 samples of large extracellular vesicles isolated from porcine seminal plasma that were used for RNA sequencing

**Additional file 4** (.xlsx). **Table S1**. Raw data for the RNAs identified in each of 12 samples of small (S) and 12 samples of large (L) extracellular vesicles isolated from porcine seminal plasma. **Table S2**. The mean percentage of read counts of each of the RNA class identified in each of 12 samples of small (S) and 12 samples of large (L) extracellular vesicles isolated from porcine seminal plasma. **Table S3**. Data of selected all RNAs, including protein-coding RNAs (mRNAs), transfer RNAs (tRNAs), ribosomal RNAs (rRNAs), small nucleolar RNAs (snoRNAs), spliceosomal U small nuclear RNAs (snRNAs), and other non-coding RNAs (ncRNAs) in each of 12 samples of small (S) and 12 samples of large (L) extracellular vesicles isolated from porcine seminal plasma. **Table S4**. Data of microRNAs identified in small (S, *n* = 12) and large (L, *n* = 12) extracellular vesicles samples isolated from porcine seminal plasma. Those with at least 10 counts in three samples were selected for further analysis. **Table S5**. Data of PIWI-interacting RNAs identified in small (S, *n* = 12) and large (L, *n* = 12) extracellular vesicles samples isolated from porcine seminal plasma. **Table S6**. Data of all RNAs differentially abundant between small (S, *n* = 12) and large (L, *n* = 12) extracellular vesicle samples isolated from porcine seminal plasma. All RNAs includes protein-coding RNAs (mRNAs), transfer RNAs (tRNAs), ribosomal RNAs (rRNAs), small nucleolar RNAs (snoRNAs), spliceosomal U small nuclear RNAs (snRNAs), and other non-coding RNAs (ncRNAs). **Table S7**. Differentially abundant microRNAs (miRNAs) between samples of small (S, *n* = 12) and large (L, *n* = 12) extracellular vesicles isolated from porcine seminal plasma. **Table S8**. List and sequencing data of putative non-microRNAs after analyzing the nucleotide sequence using the Basic Local Alignment Search Tool (BLAST) software. **Table S9**. Data of the Network of differentially abundant microRNAs (miRNAs) and overrepresented functional categories. Analysis was performed with ToppCluster software (https://toppcluster.cchmc.org/)

**Additional file 5** (.tiff). **Figure S3**. Network of overrepresented functional terms with corresponding genes for differentially abundant “all RNAs” in small (S) and large (L) porcine seminal plasma extracellular vesicles (EVs)

**Additional file 6** (.xlsx). Comparison of microRNAs (miRNAs) identified in porcine seminal extracellular vesicles in other research studies, showing those shared with the present study

## Data Availability

The datasets supporting the conclusions of this article are included within the article, in its additional files and available in at the following URL (http://www.ncbi.nlm.nih.gov/bioproject/928243; acn PRJNA928243).

## Abbreviations

AI: Artificial insemination
CFSE: Carboxyfluorescein succinimidyl ester
DA: Differentially abundant
DEGs: Differentially expressed genes
DLS: Dynamic light scattering
EVs: Extracellular vesicles
FDR: False discovery rate
FITC: Fluorescein isothiocyanate
FSC: Forward scatter
HSP: Heat shock protein
L-sEVs: Large seminal extracellular vesicles
mRNA: Messenguer RNA
miRNAs: microRNAs
ncRNA: Non-coding RNA
PBS: Phosphate buffered saline
PE: Phycoerythrin
piRNAs: PIWI interacting RNAs
PIWI: P-element-induced wimpy testes
rRNA: Ribosomal RNA
RT: Room temperature
S-sEVs: Small seminal extracellular vesicles
SEC: Size exclusion chromatography
sEVs: Seminal extracellular vesicles
snoRNA: Small nucleolar RNA
snRNA: Spliceosomal U small nuclear RNA
SP: Seminal plasma
SSC: Side scatter
TEM: Transmission electron microscopy
tRNA: Transfer RNA
