# Supplementary figures and images for: RNA profiles differ between small and large extracellular vesicle subsets isolated from porcine seminal plasma
Source: BMC Genomics. 2024 Dec 27;25:1250. doi: 10.1186/s12864-024-11167-4 (PMC11673705; doi:10.1186/s12864-024-11167-4)

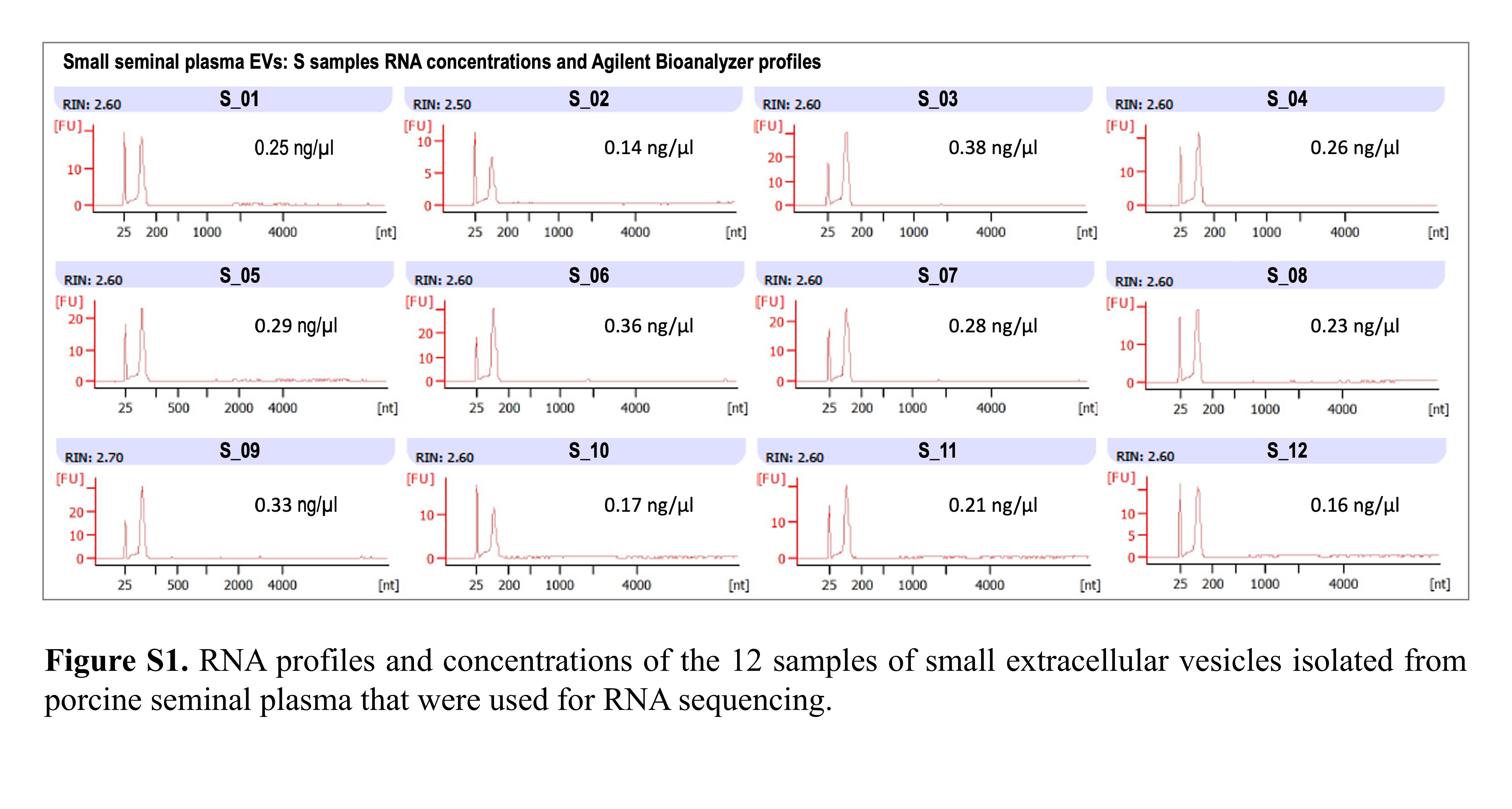

Supplement: Supplementary file 2 — Additional file 2 (.ppt). Figure S1. RNA profiles and concentrations of the 12 samples of small extracellular vesicles isolated from porcine seminal plasma that were used for RNA sequencing [file 12864_2024_11167_MOESM2_ESM.tif]

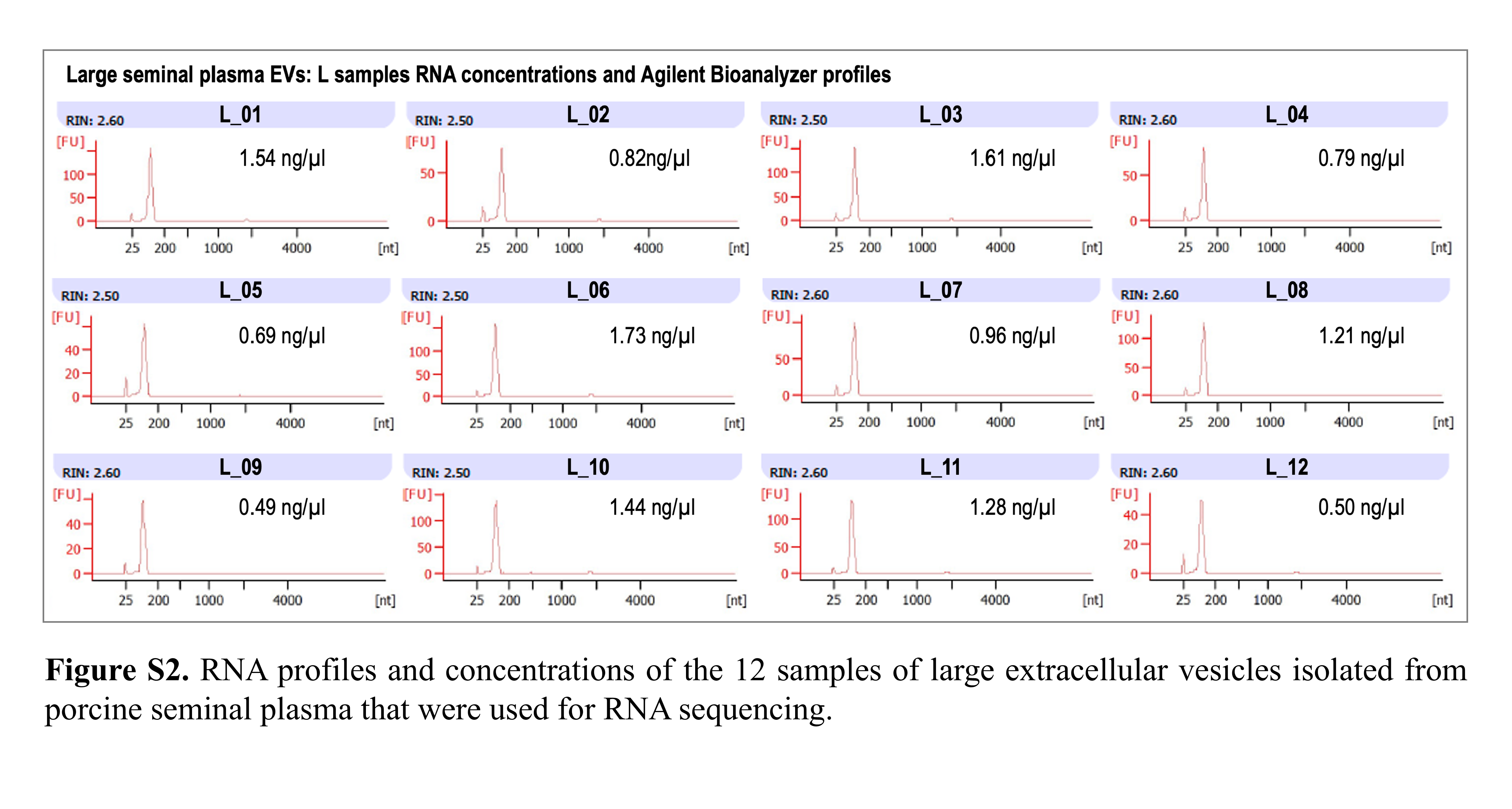

Supplement: Supplementary file 3 — Additional file 3 (.ppt). Figure S2. RNA profiles and concentrations of the 12 samples of large extracellular vesicles isolated from porcine seminal plasma that were used for RNA sequencing [file 12864_2024_11167_MOESM3_ESM.tif]

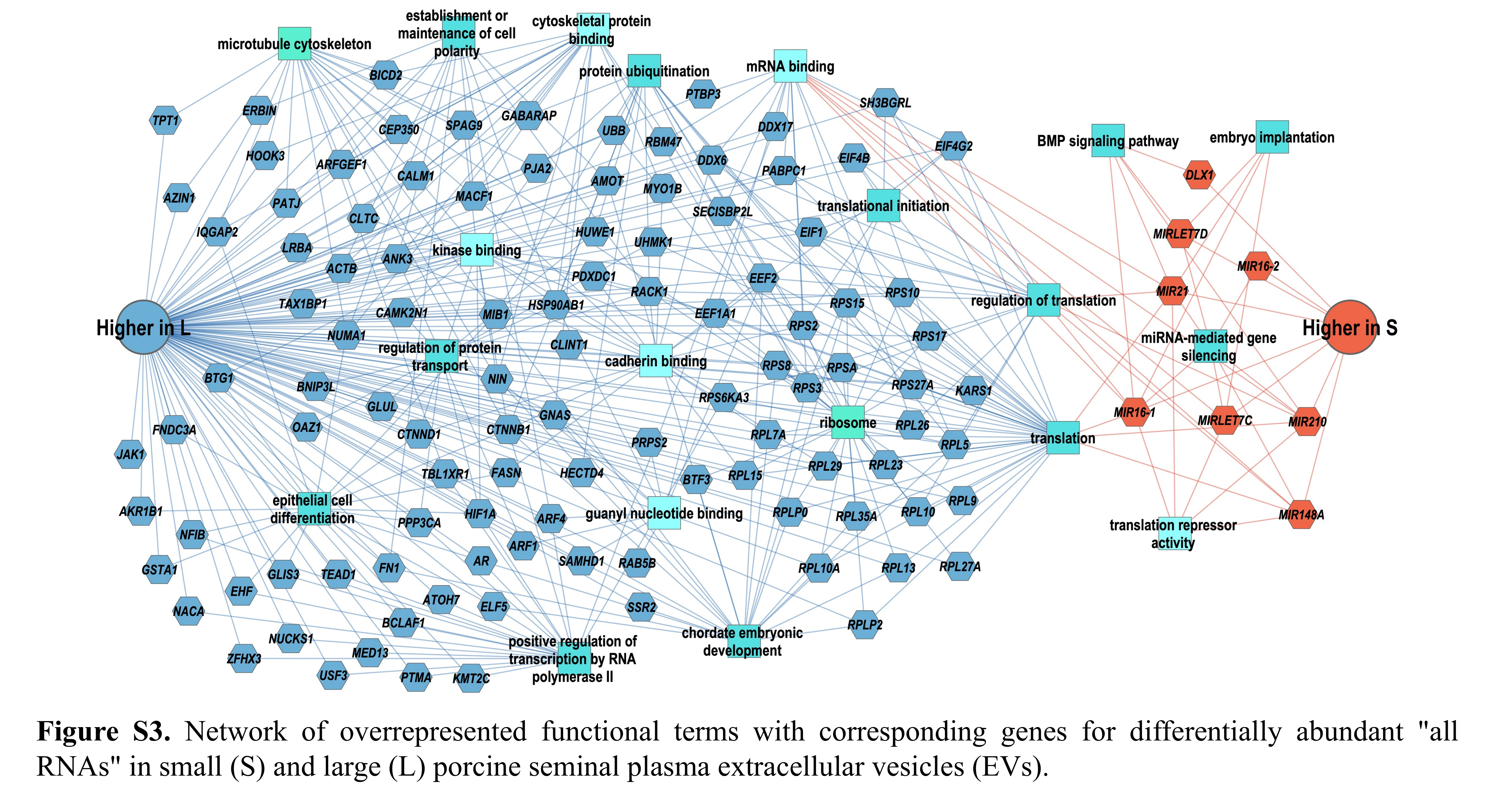

Supplement: Supplementary file 5 — Additional file 5 (.tiff). Figure S3. Network of overrepresented functional terms with corresponding genes for differentially abundant “all RNAs” in small (S) and large (L) porcine seminal plasma extracellular vesicles (EVs) [file 12864_2024_11167_MOESM5_ESM.tif]
